# Automated morphometrics on microscopy images of Atlantic cod larvae using Mask R-CNN and classical machine vision techniques

**DOI:** 10.1016/j.mex.2021.101598

**Published:** 2021-12-06

**Authors:** Bjarne Kvæstad, Bjørn Henrik Hansen, Emlyn Davies

**Affiliations:** SINTEF Ocean, Environment and New Resources, Brattørkaia 17C, Trondheim NO-7010, Norway

**Keywords:** Morphometrics, Ecotoxicity, Microscopy, Machine learning, Artificial neural networks, Instance segmenting, Machine vision

## Abstract

Measurements of morphometrical parameters on i.e., fish larvae are useful for assessing the quality and condition of the specimen in environmental research or optimal growth in the cultivation industry. Manually acquiring morphometrical parameters from microscopy images can be time consuming and tedious, this can be a limiting factor when acquiring samples for an experiment. Mask R-CNN, an instance segmentation neural network architecture, has been implemented for finding outlines on parts of interest on fish larvae (Atlantic cod, Gadus morhua). Using classical machine vision techniques on the outlines makes it is possible to acquire morphometrics such as area, diameter, length, and height. The combination of these techniques is providing accurate-, consistent-, and high-volume information on the morphometrics of small organisms, making it possible to sample more data for morphometric analysis.

• Capabilities to automatically analyse a set of microscopy images in approximately 2-3 seconds per image, with results that have a high degree of accuracy when compared to morphometrics acquired manually by an expert.
• Can be implemented on other species of ichthyoplankton or zooplankton and has successfully been tested on ballan wrasse, zebrafish, lumpsucker and calanoid copepods.

## Graphical abstract

**Figure:**
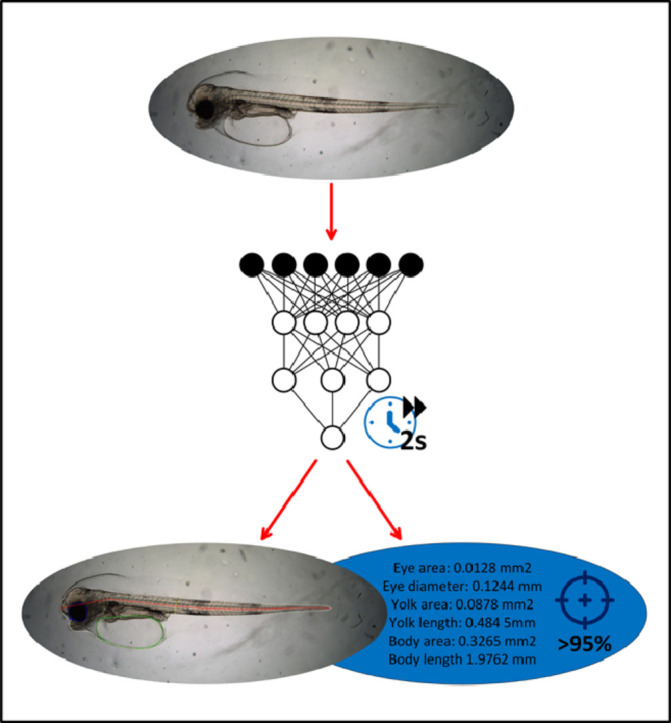

Specifications Table

**Table:** 

Subject Area:	Computer Science
More specific subject area:	*Biology; morphometrics*
Method name:	*AutoMOMI (Automated Morphometrics On Microscope Images)*
Name and reference of original method:	*n/a*
Resource availability:	https://doi.org/10.5281/zenodo.5745209

## Method details

AutoMOMI (Automated Morphometrics On Microscope Images) provides a framework for automated morphometrical analysis of microscopy images, using Mask R-CNN [9], written in Python 3.6. Mask R-CNN is an instance segmentation neural network architecture developed by Facebook AI Research (FAIR), which has been trained to identify and outline parts of interest on the microscopy image. The morphometrical measurements, like area and length on Atlantic cod, are acquired from the Mask R-CNN outlines using classical machine vision techniques. This article will discuss the implementation of this method on larvae of Atlantic cod, but it can also be implemented on other species of ichthyoplankton or zooplankton.

## Automated morphometrical analysis

**Fig. 1:**
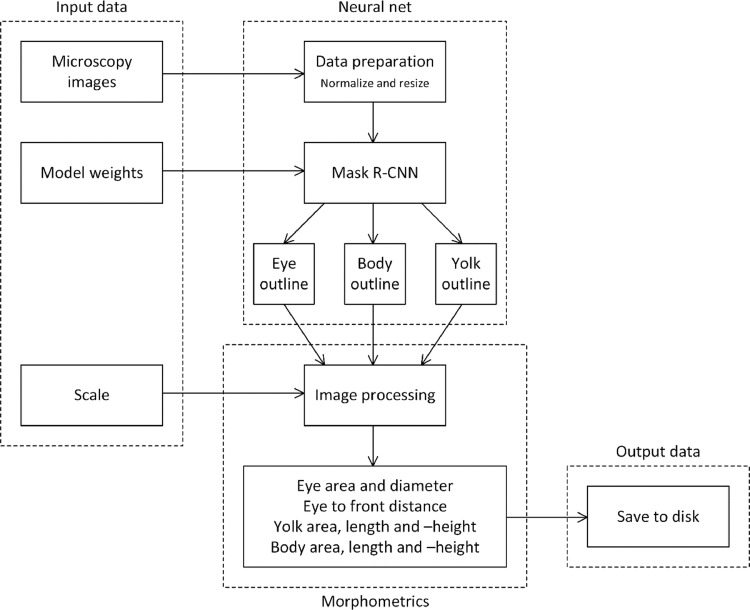
A flow diagram of the automated process for morphometric analysis, with its four main modules: input data, neural network, morphometrics and output data.

Figure 1 presents an overview of the automated data processing flow, from raw microscope images to morphometric parameters represented in a human-readable data file. The four main modules, (i) input data, (ii) neural net, (iii) morphometrics and (iv) output data are explained in detail in this section.

### Input data

Executing the processing algorithm requires: (i) Model weights, a H5 binary file containing the weights that is applied to the neural net architecture to detect the correct features in the input image. The weights file is derived from training the neural network and is explained in section 2. (ii) A scaling parameter, in pixels per millimetre, this parameter can vary depending on the optics used on the microscope or the resolution of the image sensor (usually acquired by imaging a scale bar). (iii) The microscopy images to be processed in a JPG-, JPEG-, PNG- or BMP image format. A framework has been developed that will automatically process microscope images from a folder, this way the algorithm can process a batch of microscopy images completely unattended.

### Neural net / Mask R-CNN

**Fig. 2:**
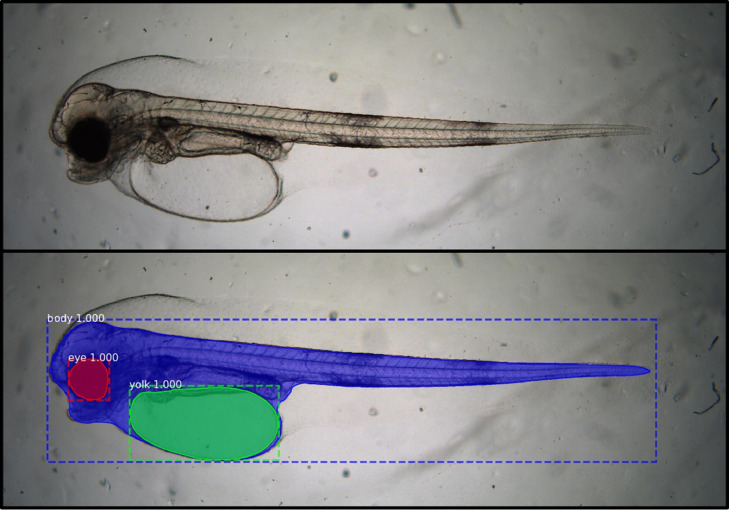
An illustration of the Mask R-CNN neural network output data (bottom) from an input image (top), where the body (blue), eye (red) and yolk sac (green) is outlined and marked with the confidence value from the neural network, in this case all detected parts of interest had a confidence value of 1.0 (100%).

The neural network has a fixed input, meaning all microscopy images must have a specified size, therefore the images need to be rescaled to a size of 1024 × 1024 pixels. To speed up training the input image is normalized around pixel value *123.7* (Red), *116.8* (Green) and *103.9* (Blue), so that the model weight parameters can converge closer to zero mean. Matterport's implementation of Mask R-CNN [1] was used for AutoMOMI as it contains good documentation for implementing a custom data set. The neural network output is a *K* x *N* x *M* matrix containing *K* binary masks with the same dimension as the input image (*N* x *M*), along with a Region of Interest (ROI) for each mask. Each binary mask (*K*) represents the segmented area of each detected object, where the pixel values can be "1" (segmented area) or "0" (background), the ROI and binary masks are illustrated in Figure 2.

### Morphometrics

The morphometrics are based on the masks generated from the neural network output and the scaling parameter. Extracting the morphometrics, i.e. length and area, is achieved with classical machine vision techniques using OpenCV v4.1 [2] and Scikit-Image v0.16.2 [14] in Python (Table 1).

**Table 1:** Explanation of how each morphometric parameter is obtained from the binary masks

Yolk & Eye		**Area** The area (blue), is calculated by summing the binary pixel values in the binary mask and multiply with the square of the scaling parameter. The same technique is used for calculating body area (white). **Diameter** Min/max (green/red) diameter is derived using the Scikit-Image functions, "label" and "regionprops" to acquire the minor- and major axis of a least square fitted ellipse to the eye and yolk mask. Multiplying with the scaling parameter gives minimum and maximum diameter of the eye and yolk. **Eye to front** Minimum distance from eye to front end (orange) is used as an indicator of the degree of craniofacial deformation of the cod larvae. This metric is calculated by finding the shortest path from the frontal edge of the eye to the forehead.
Body		**Myotome height** The myotome height (yellow) is the myotome cross section measured, on the body mask, from right behind the anus multiplied with the scaling factor. Using the body mask, we can calculate an approximate body orientation by defining a 100 pixels long line on the body outline, centred above the myotome cross section. Calculating the angle of this line we can adjust the cross section according to the orientation of the larvae, as the cross section should be orthogonal to the body orientation. **Standard length** The standard length (red) is the length measured from forehead along the myotome to the end of the notochord, multiplied with the scaling factor. The tail is measured using Scikit function "skeletonize" [15] and the preanal body area is measured by finding the distance from the yellow line (myotome height) to the forehead via the neck.

### Output data

**Fig. 3:**
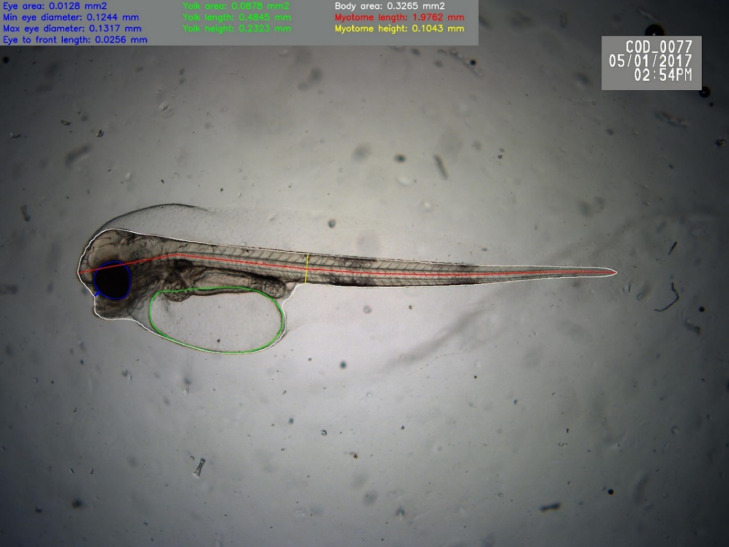
An automatically rendered microscopy image of an Atlantic cod larvae (3 days post fertilization) with the morphometrics for manual verification of body area (white line), yolk sac area (green line) and eye area (blue line), as well as standard length (red line), forehead to eye distance (blue line) and myotome height (yellow line). The morphometric parameters is automatically added to the top left corner.

The framework built around the automated morphometrics algorithms will automatically acquire the microscopy images from a folder for analysis. The morphometrics acquired from AutoMOMI is stored in a CSV file (Comma Separated Values) containing data for every microscopy image processed from the folder, making it possible to import to Excel, MATLAB or other software for statistical analysis. To make the analysed data trustworthy, the algorithm will automatically render the calculated morphometrics on top of the microscopy image (Figure 3) for verification by expert eye.

## Training the neural network

**Fig. 4:**
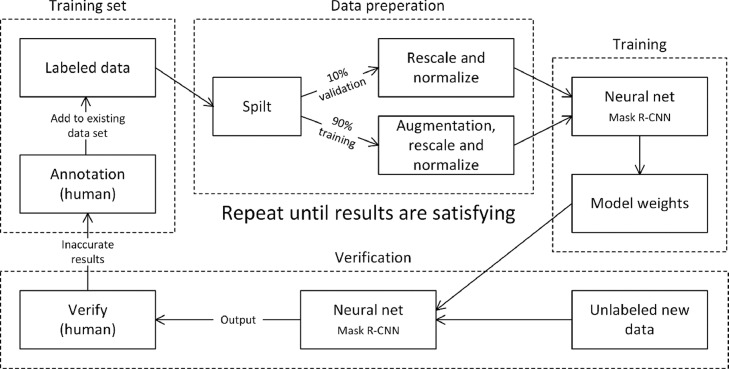
A flow diagram of the procedure for training the neural network for predicting outlines for a new species. The procedure is spilt in to four modules: training set, data preparation, training, and verification. This process is repeated until the end results are satisfactory.

To avoid potential accuracy loss due to an imbalanced training set [10, 12], the different species are trained separately, this approach gives one set of model weights per species and will not be compatible with each other. Species classification is not the main objective for AutoMOMI, and it is expected that the user already has categorized the species beforehand, as a part of the procedure for implementing support for a new species. Figure 4 illustrates the process flow for implementing a new species to the AutoMOMI framework by training the Mask R-CNN neural network.

### Training set

ImageJ [13] was used for manually acquiring measurements on microscope images before AutoMOMI was developed, meaning that 122 images of Atlantic cod with outlined yolk sacs was already available from a previous experiment published in Hansen *et al.* [5]. The outline data are stored in the metadata of a TIF (Tagged Image File Format) file along with the respective images, a Python script was therefore developed to extract the metadata in the TIF file to a CSV file format along with the images stored as JPGs. The CSV file has the same format as VIA (VGG Image Annotator), a lightweight open-source software for annotation of JPG-, JPEG-, BMP- and PNG image file formats [3]. Meaning that AutoMOMI can be trained on data annotated with ImageJ and VIA, however only ImageJ was used due to the familiarity for the biologist annotating the data.

From the 122 images, 48 of the images got the remaining body- and eye outline annotated, serving as the initial training set for Atlantic Cod. Although 48 images in the initial training set is conserdered as small, it turned out to give an average accuracy of more than 90% when testing on images from the same batch. This was due to images in this batch were taken from the same microscope, containing exactly one larva with roughly the same background. However, the dataset was not sufficient when analysing a different batch of images, captured under circumstances like different optics, -lighting or -larvae age/size. Therefore, over a two-year period, the Atlantic Cod training set has grown to 213 images trough two additional training iterations, using the method described in Figure 4. By assessing the rendered output image (Figure 3), the worst performing images per new batch of images was annotated and appended to the existing training set, 58 and 109, respectively. Over time, building a larger and more diverse training set that will increase the overall accuracy as the neural net starts to generalize better, as the training set grows, it is expected that the accuracy will at some point plateau and appending new training data will no longer be necessary [12].

### Data preparation

Of the total dataset, 90% is used for training, and the remaining 10% is used for validation, both is normalized and resized in preparation of training. To expand the training set further image augmentation is applied using imgaug [11], an open source Python based platform for augmenting training set for machine learning. Using the imgaug function “SomeOf”, a random selection between 1 and 5 from the list augmenters below was used on the training set for each training step.

1. 50% probability of flipping image horizontally (“Fliplr”)
2. Image channel multiplication of a random value between 0.7 and 1.3 (“Multiply”)
  a. 75% probability of per channel multiplication
  b. 25% probability of image wise multiplication
3. One of the following (“OneOf”)
  a. Gaussian blur with a random kernel sigma value between 0.0 and 5.0 (“GaussianBlur”)
  b. Simplex noise alpha with Gaussian blur with a kernel sigma of 10.0 (“SimplexNoiseAlpha”)
4. Rotate image by a random value between -45.0 and 45.0 degrees (“Affine”)
5. Shear image by a random value between -25 and 25 degrees (“Affine”)
6. Warp the image by a random scale between 0.01 and 0.05 (“PiecewiseAffine”)
7. Crop and pad the image by a random value between 0 and 300 pixels on the horizontal axis and 0 and 100 pixels on the vertical axis (“CropAndPad”)

Choosing the right augmentors with its parameters was determined by selecting realistic parameters that make the image look similar to the original data, but with a slight variation. E.g., the larvae direction can vary, parts of- or the whole image can be slightly out of focus, background colour and brightness can vary and so on. However, the larvae will never be imaged upside-down, or be so out of focus that not even an expert could classify it, hence flipping the image upside-down or applying Gaussian blur with a too high kernel sigma would be unrealistic.

### Training

Using the existing framework in the Matterport source code, the neural network was trained on a desktop computer (Ubuntu 18.04, Nvidia RTX 2080Ti, Intel i9-9900k, 32Gb RAM). During training, loss metrics were logged to Tensorboard and a snapshot of the model weights was saved to disk after every epoch, approximately every 7^th^ minute of training. The training was stopped when the validation loss started to increase relative to the training loss (overfitting), after 1028 epochs. By training the neural net until it starts to overfit, we are sure that the neural net accuracy will not improve with more training. Using loss metrics from Tensorflow the model weights from epoch 679 was selected as it had the lowest validation loss.

### Verification

A total of 1372 unannotated microscopy images of Atlantic cod larvae were analysed using the neural network, the results were manually verified by an expert by inspecting the automatically rendered images (Figure 3), any misclassified image were annotated and appended to the existing training set for further training of the neural network. This process was repeated until the number of rejected images due to misclassification was less than 10%, and after an additional 58 images of cod was appended to the training set.

## Method validation

**Fig. 5:**
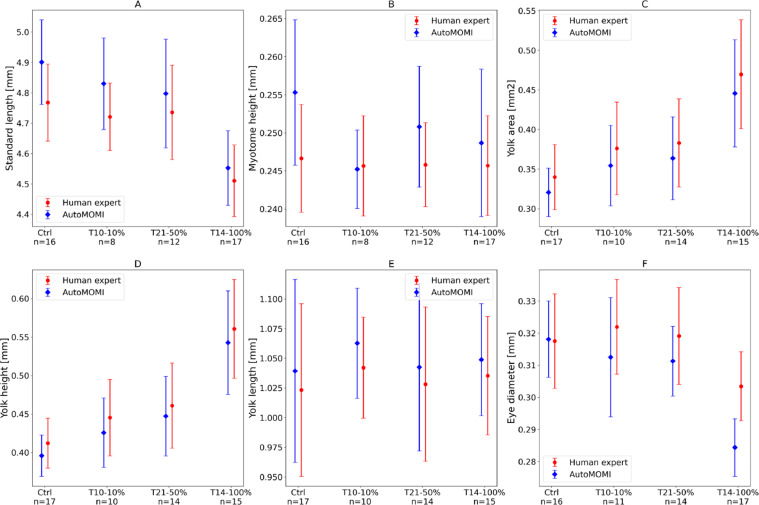
A side-by-side comparison plot for morphometrics acquired automatically with AutoMOMI (blue) and manually from an expert (red) for each exposure group (x-axis) and end point (sub plots).

AutoMOMI was validated with a dataset from an experiment where Atlantic cod embryos were exposed to crude oil resulting in morphological and developmental deformations [5]. The dataset contained 77 images of larvae of which 18 larvae were negative controls (treated with filtered sea water), 11 larvae exposed to water-soluble fractions (WSF) of crude oil degraded for 10 days diluted to 10% (T10-10%), 14 larvae exposed to WSF degraded for 21 days diluted to 50% (T21-50%) and 18 larvae exposed to WSF biodegraded for 14 days undiluted (T14-100%), all images were taken of larvae 3 days post hatch. Morphometrical data (standard length, body area, myotome height, eye diameter and yolk sac area) from the experiment had manually been acquired by an expert in the field. These measurements were compared with the data acquired using AutoMOMI, with model weights trained on a different dataset containing 213 images of Atlantic cod. By manually analysing the rendered images (Figure 3), 44 measurements (out of 770) were removed due to misclassifications, this includes width, height and area of 6 yolk sacs, 9 bodies and 4 eyes. In order to compare the results from AutoMOMI and the expert, the average and standard deviation of each endpoint was calculated for each experiment group and plotted side-by-side (Figure 5).

**Fig. 6:**
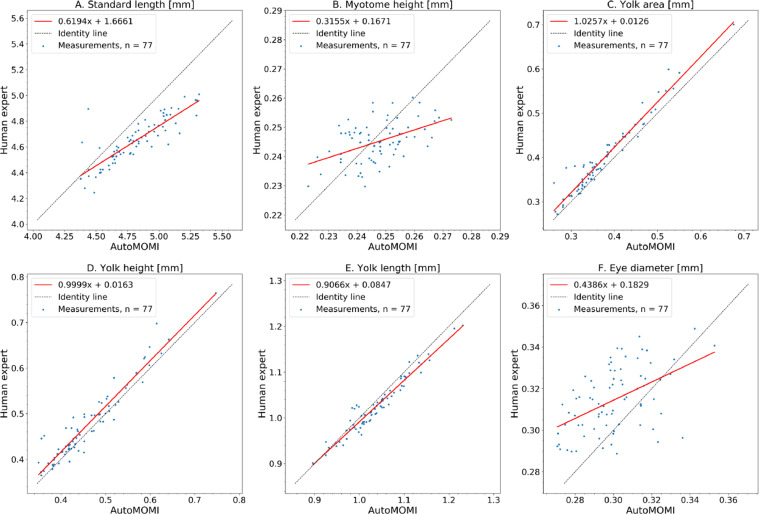
A scatter plot with linear regression, where measurements from a human expert (y axis) are compared with measurements from AutoMOMI (x axis) with a best fit regression line (red) and a linear line with a slope number of 1 (dotted black).

When inspecting Figure 5, the data from both methods are comparable as both are sharing the same trend, e.g., higher concentration of WFS affects the larva's ability to consume its yolk sac (Figure 5C) or does not have an effect on yolk sac length (Figure 5E). However, in the short measurements (<0.4mm) like myotome height- and eye diameter measurements (Figure 5B and -F), there is a larger deviation in trend between the results from manual acquisition and AutoMOMI when compared to the longer measurements (Figure 5A, -C, -D and -E). This highlights a possible limitation of AutoMOMI when it comes to smaller measurements on an image, this becomes more apparent when plotting the results as a scatter plot with regression line (Figure 6), the measurements for subplots B and F are more scattered and the regression line is less linear than subplots A, C, D, and E.

Another factor to consider when doing the analysis manually with ImageJ is finding the morphometric end points for 77 microscopy images is approximately a day's work, the results might also vary from person to person, time of day or mood. AutoMOMI can processes one microscopy image in approximately 2-3 seconds and the entire dataset of 77 images in less than four minutes, completely unattended.

Because of AutoMOMI we are now able to sample even more data for morphometric analysis, as we are only required to validate the automatically rendered images instead of doing the morphometric analysis manually. It is also faster to implement a new species for larger data sets than to perform the morphometric analysis of the whole data set manually, as the training set can consist of a fraction of the total data set in one experiment. So far, AutoMOMI has successfully been tested on yolk-sac larvae of Atlantic cod (*Gadus morhua*), Atlantic haddock (Melanogrammus aeglefinus), ballan wrasse (*Labrus bergylta*), zebrafish (*Danio rerio*), lumpsucker (*Cyclopterus lumpus*) and on different copepodite stages of the copepods *Calanus finmarchicus* and *Calanus glacialis* (Appendix A), as well as used in ecotoxicology research [7].

## Background

Morphometrical parameters in developing fish larvae can be used to assess larvae quality and condition. Morphometrics can provide valuable information regarding optimal rearing conditions for the cultivation industry (Fotedar, 2017) [4]. It is also extensively used, as a complementary tool to traditional time-consuming staining methodologies for assessing the presence of developmental malformations in ecotoxicological studies (Hansen et al., 2019) [8]. Morphometrics have also proven very useful in studies investigating developmental effects of pollutants on zooplankton development (Hansen et al., 2016) [6]. High-quality and standardized images of fish larvae are regularly used to measure different features, like standard length, body area, yolk sac size and eye diameter, using ImageJ, an open-source image processing program. Unfortunately, using ImageJ (or other tools) for this task (especially microscopy images with a complicated background, varying light, and random blurriness) is a time consuming and laborious manual task and can be a limiting factor when acquiring samples for an experiment.

## Declaration of Competing Interest

The authors declare that they have no known competing financial interests or personal relationships that could have appeared to influence the work reported in this paper.

## Appendix Supplementary materials

Image, application 1
