# Supplementary material for: Automated morphometrics on microscopy images of Atlantic cod larvae using Mask R-CNN and classical machine vision techniques
Source: MethodsX. 2021 Dec 6;9:101598. doi: 10.1016/j.mex.2021.101598 (PMC8666706; doi:10.1016/j.mex.2021.101598)
Supplement: Supplementary file 1 [file mmc1.docx]

Appendix A – Other species


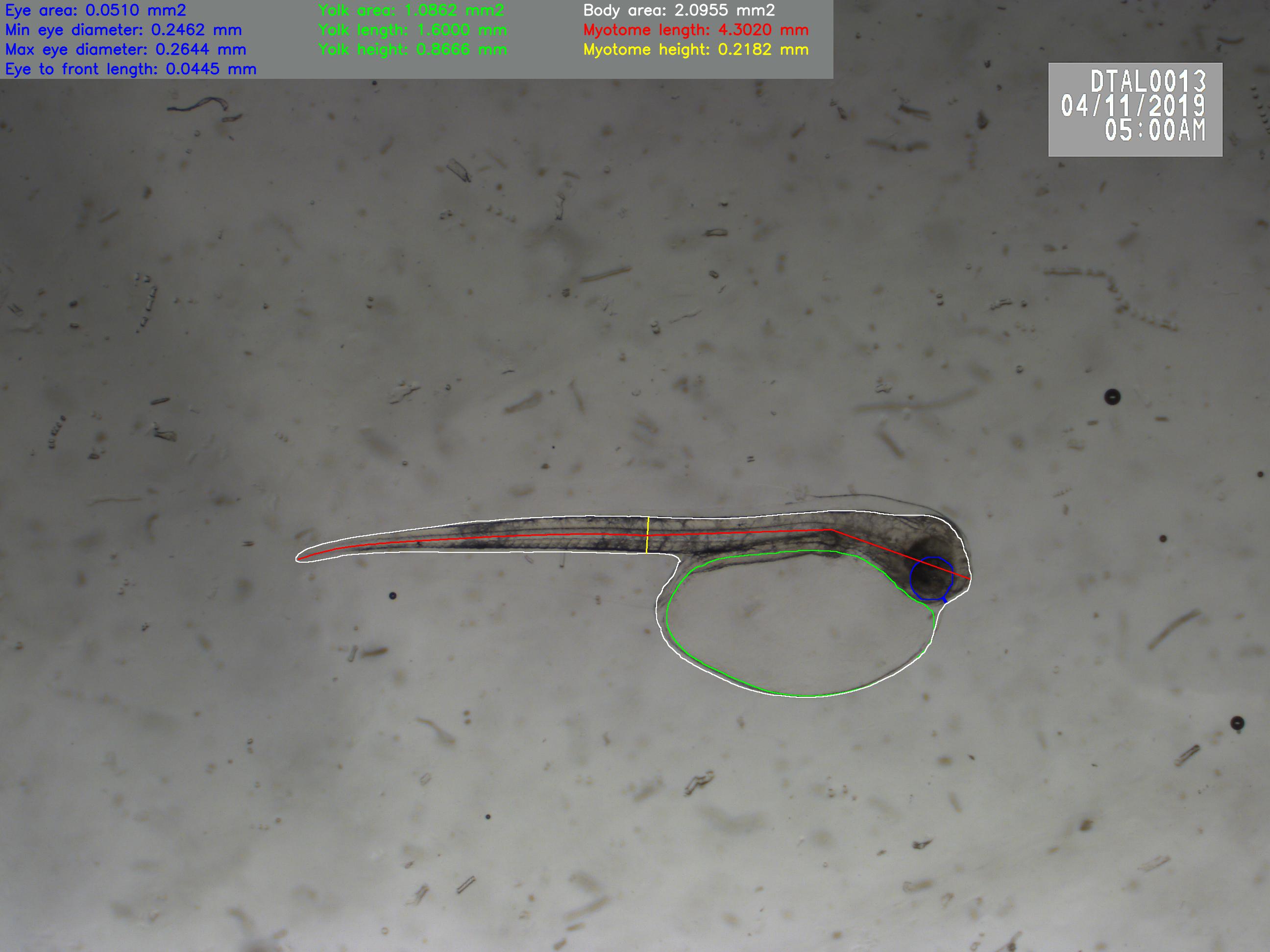


Figure 1 Atlantic cod (Gadus morhua). Foto: Julia Farkas, SINTEF Ocean


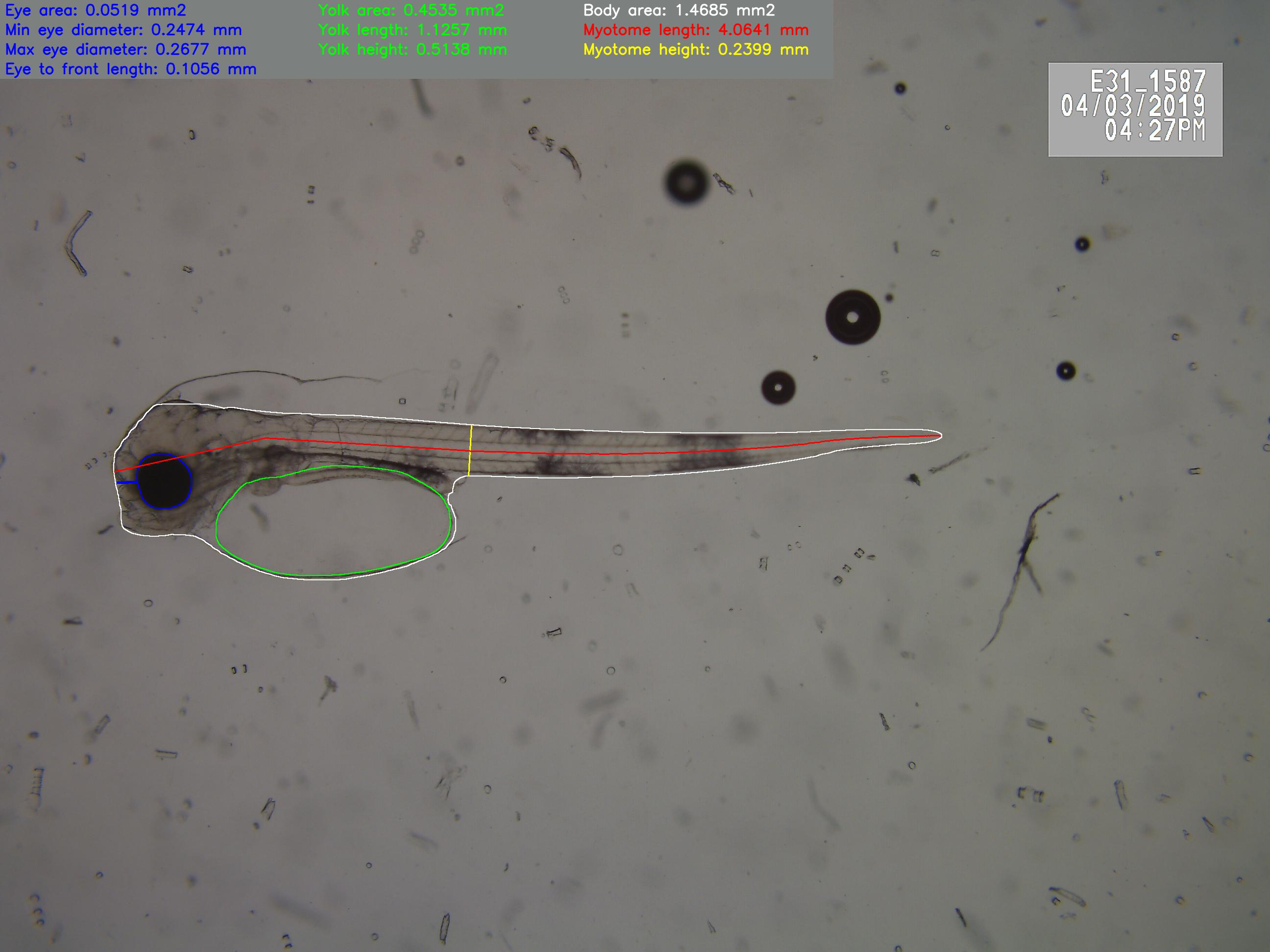


Figure 2 Atlantic cod (Gadus morhua). Foto: Bjørn Henrik Hansen, SINTEF Ocean


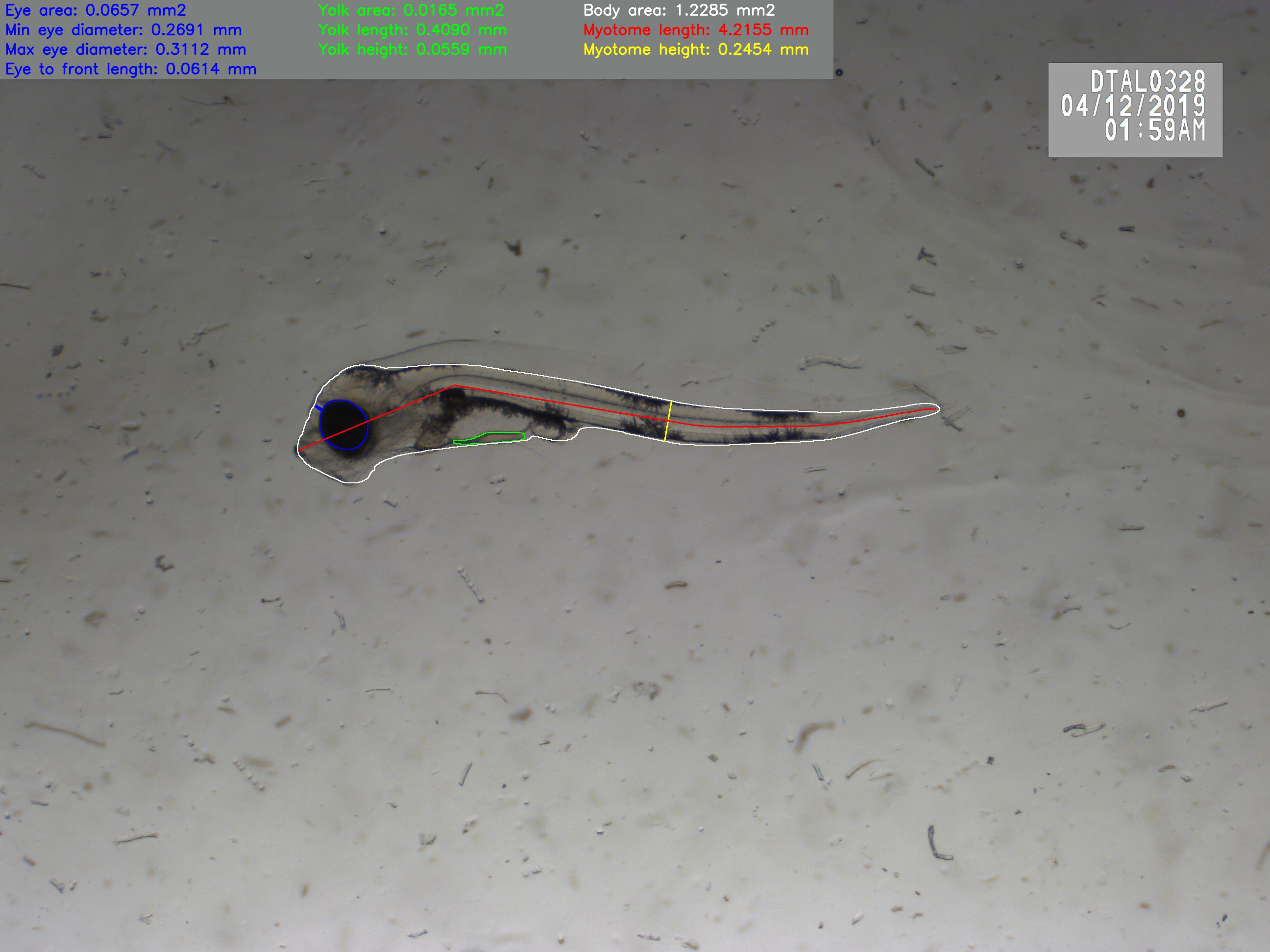


Figure 3 Atlantic cod (Gadus morhua). Foto: Julia Farkas, SINTEF Ocean


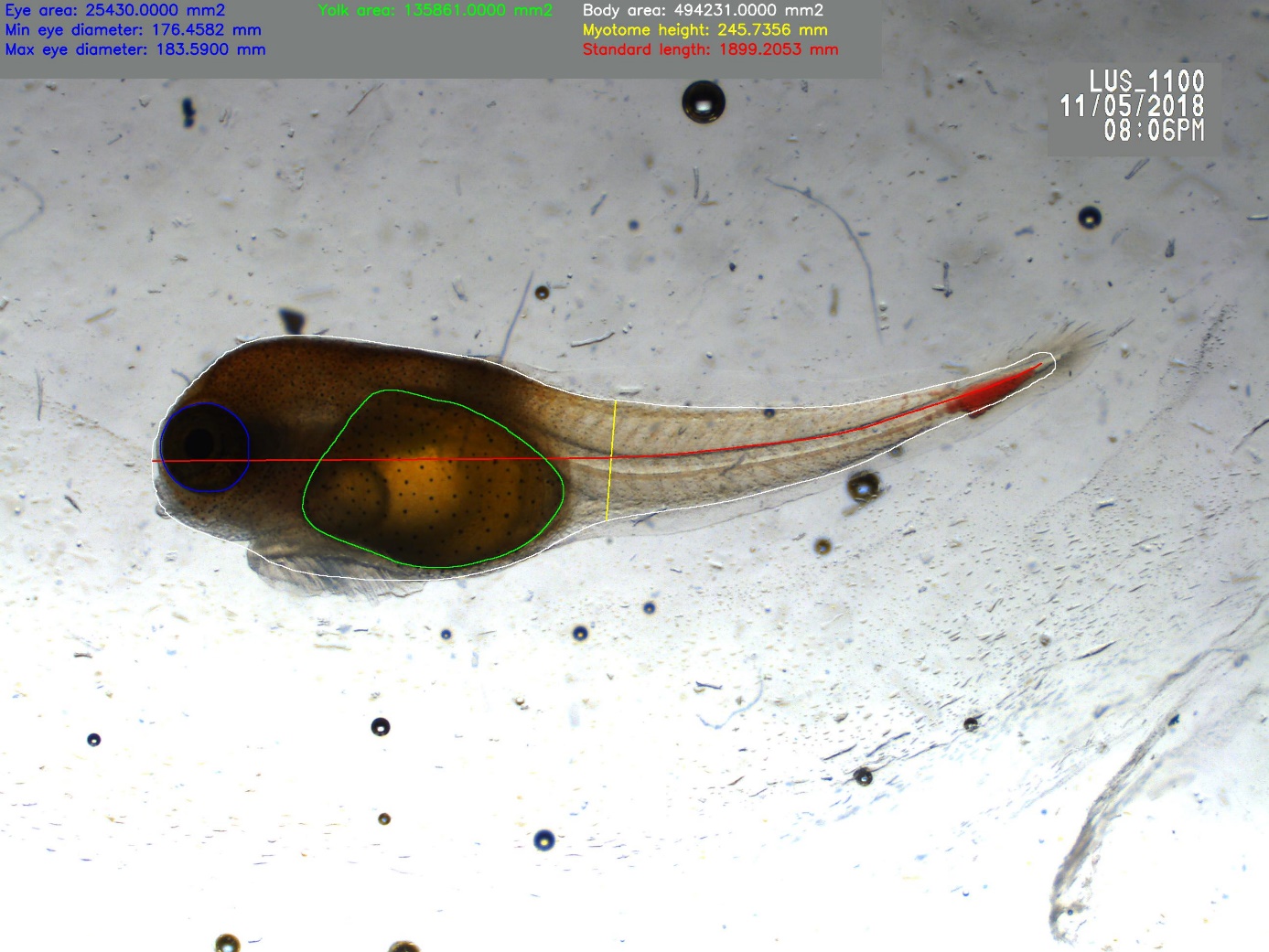


Figure 4 Lumpsucker (Cyclopterus lumpus). Foto: Bjørn Henrik Hansen, SINTEF Ocean


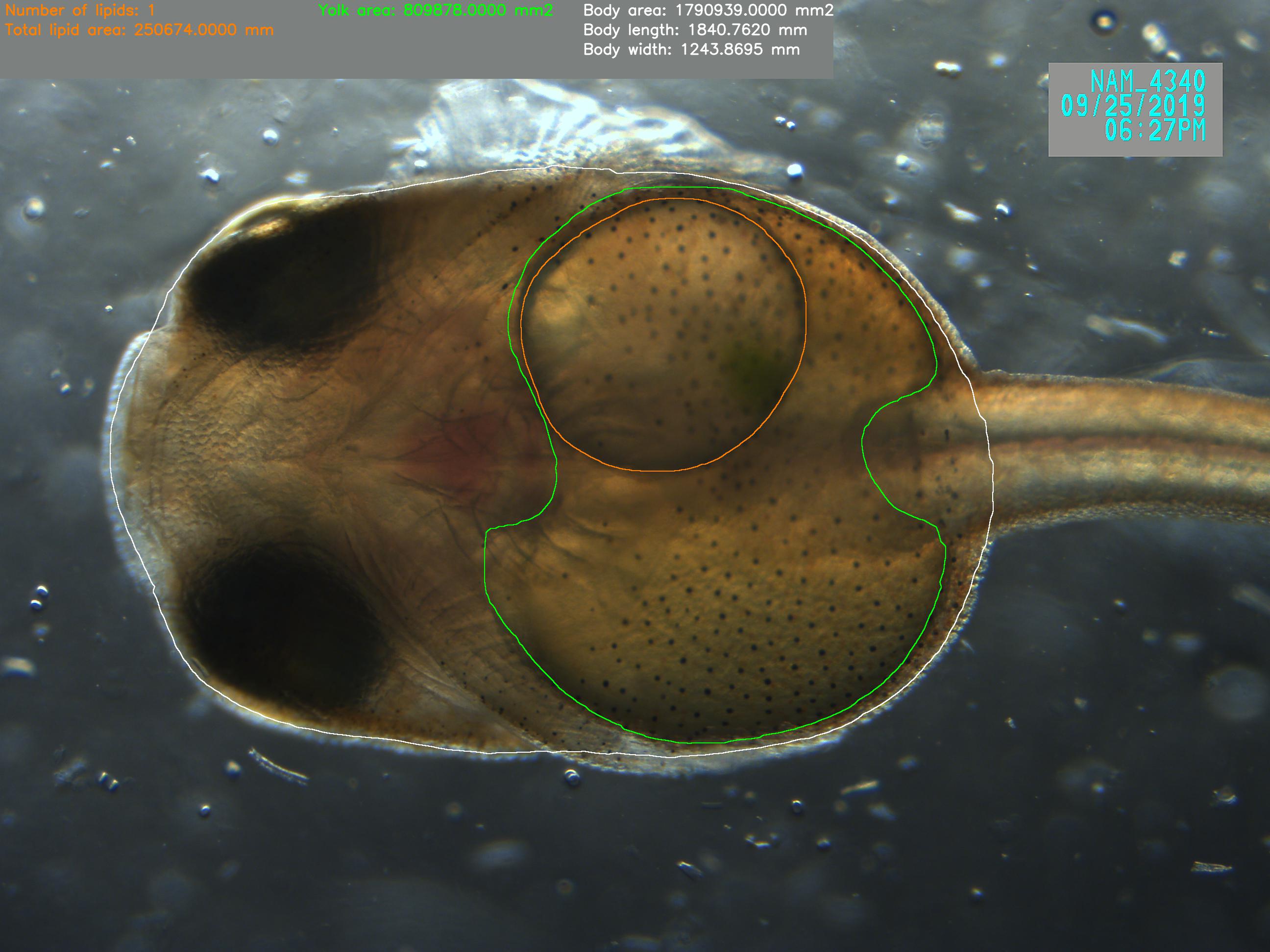


Figure 5 Lumpsucker (Cyclopterus lumpus). Foto: Bjørn Henrik Hansen, SINTEF Ocean


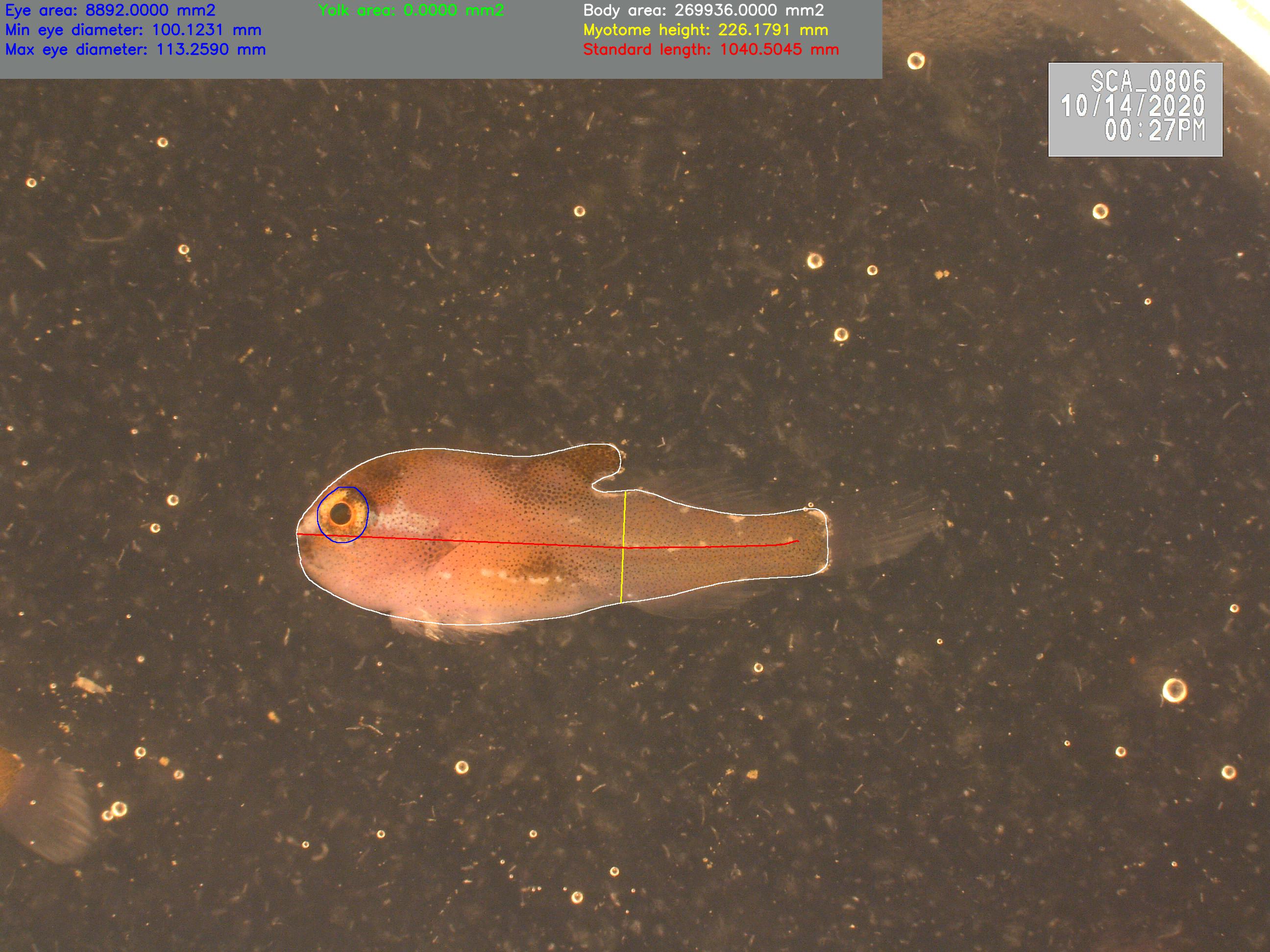


Figure 6 Lumpsucker (Cyclopterus lumpus). Foto: Bjørn Henrik Hansen, SINTEF Ocean


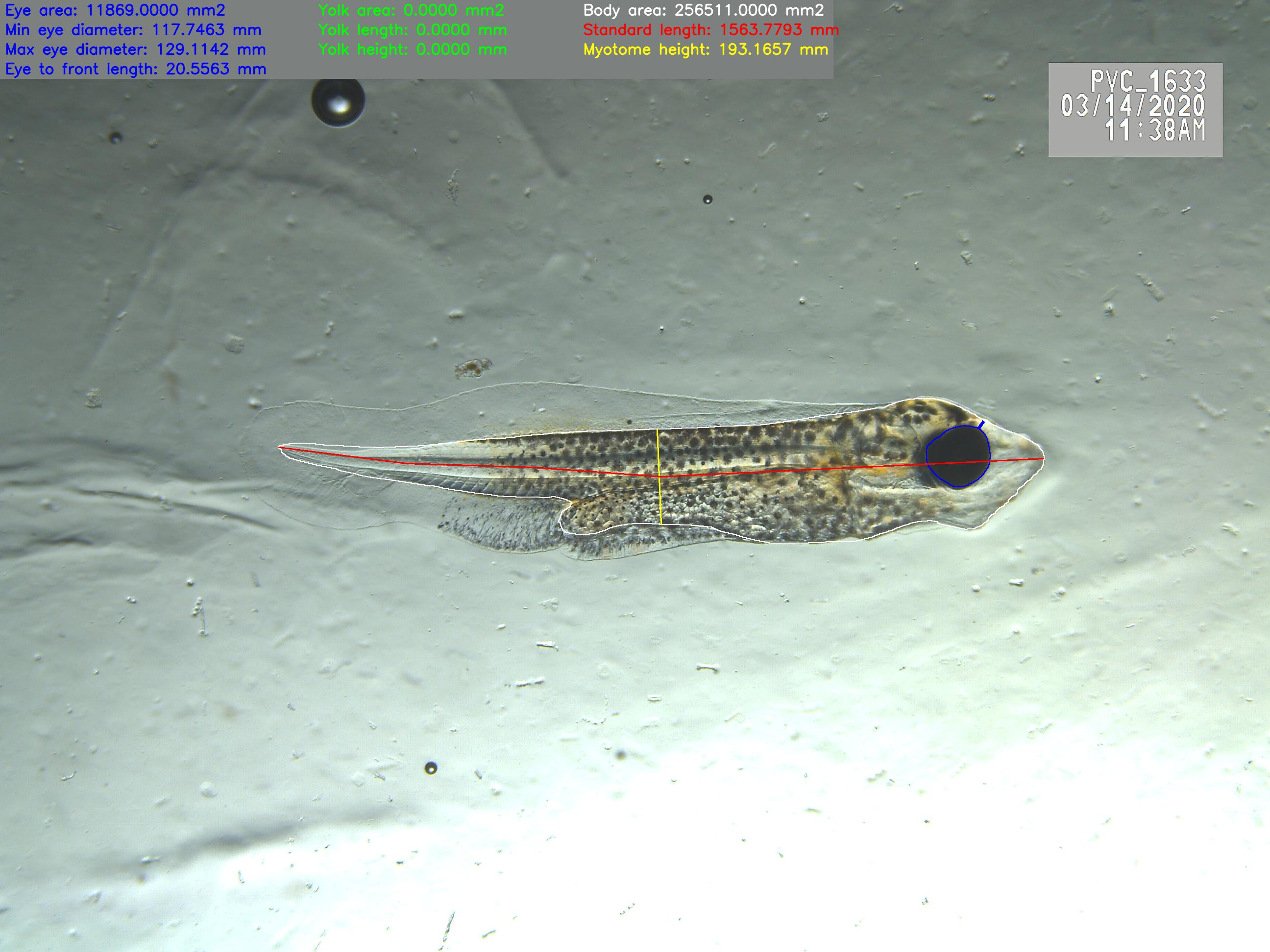


Figure 7 Ballan wrasse (Labrus bergylta). Foto: Bjørn Henrik Hansen, SINTEF Ocean


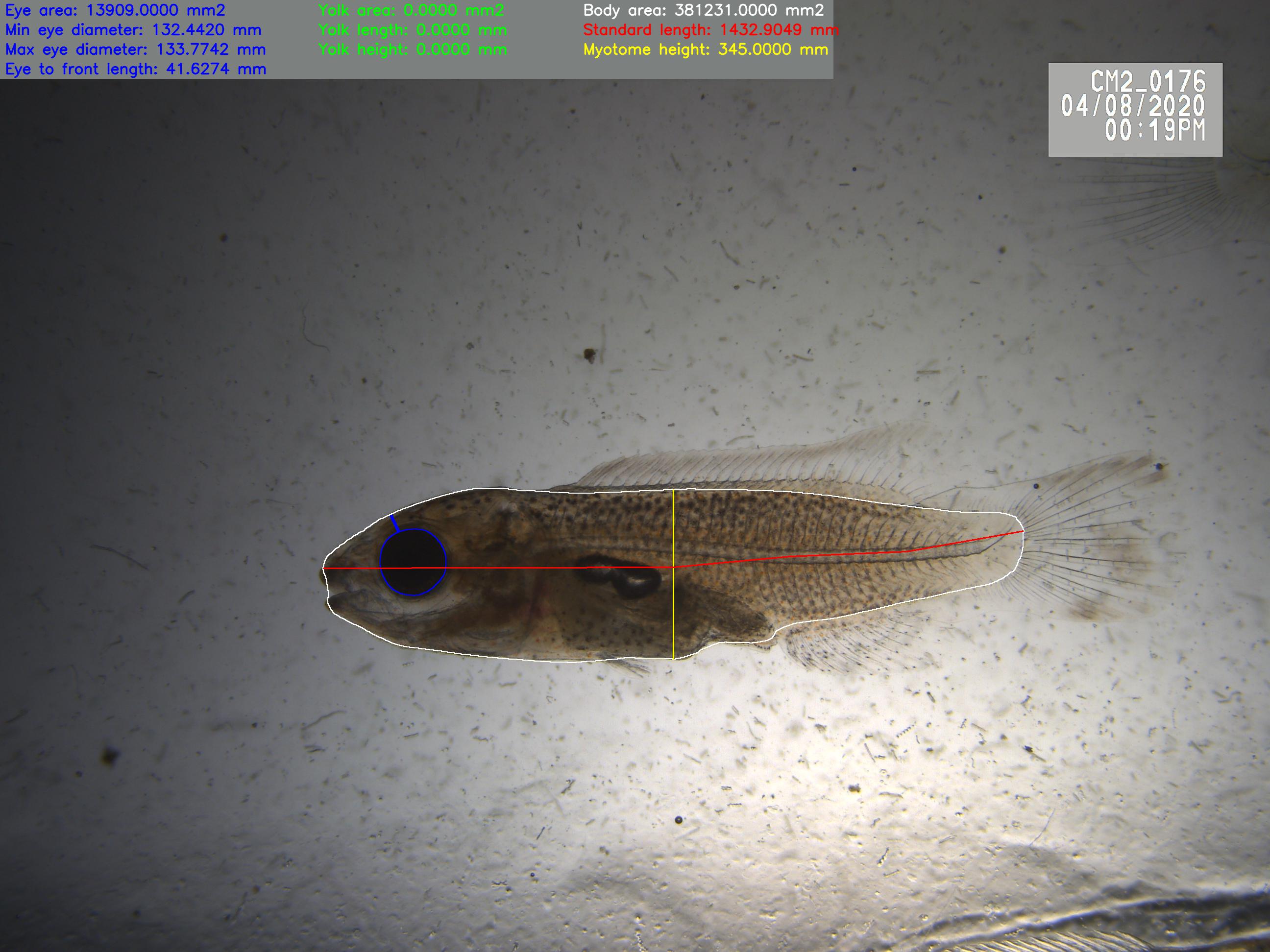


Figure 8 Ballan wrasse (Labrus bergylta). Foto: Bjørn Henrik Hansen, SINTEF Ocean


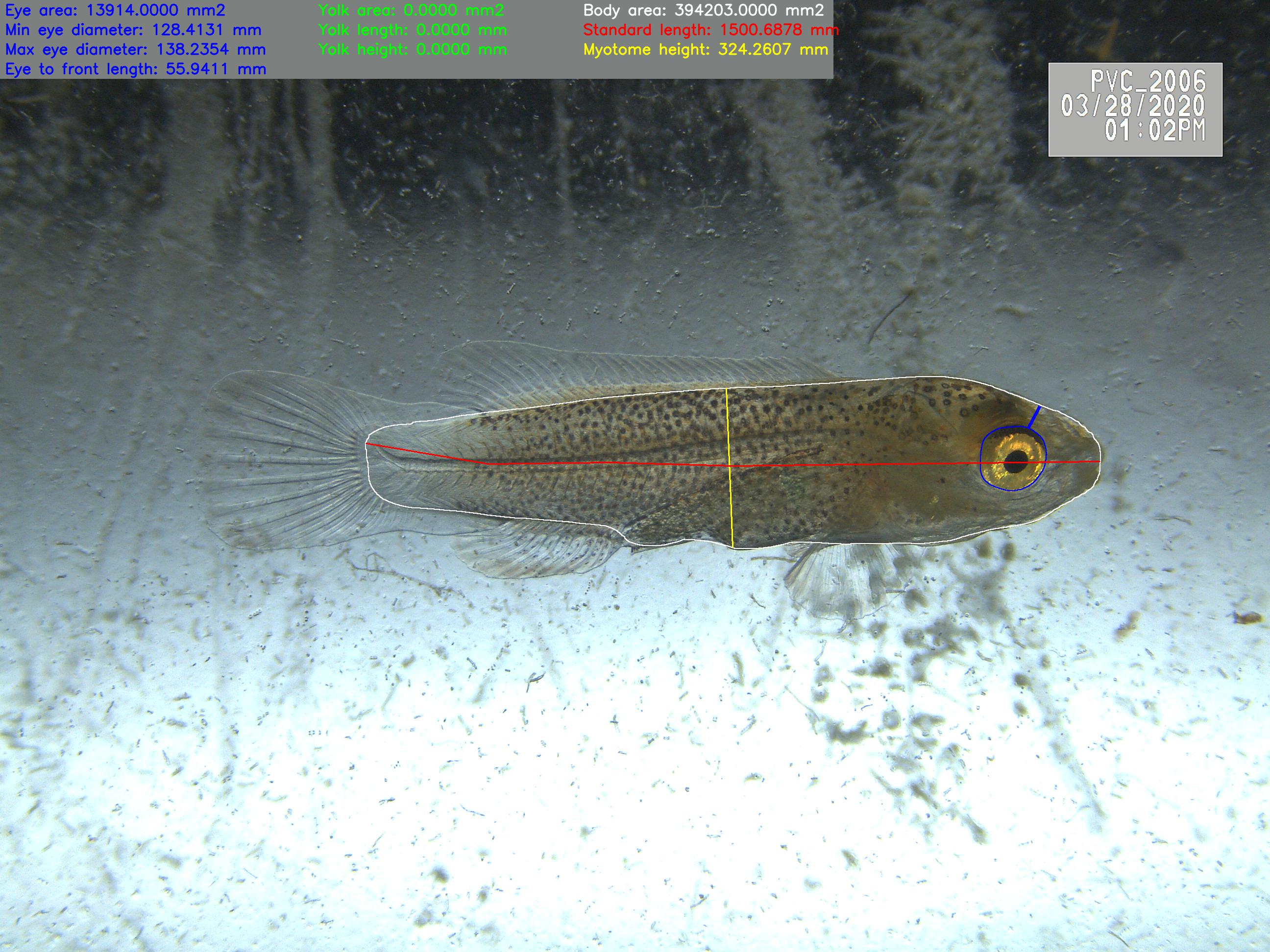


Figure 9 Ballan wrasse (Labrus bergylta). Foto: Bjørn Henrik Hansen, SINTEF Ocean


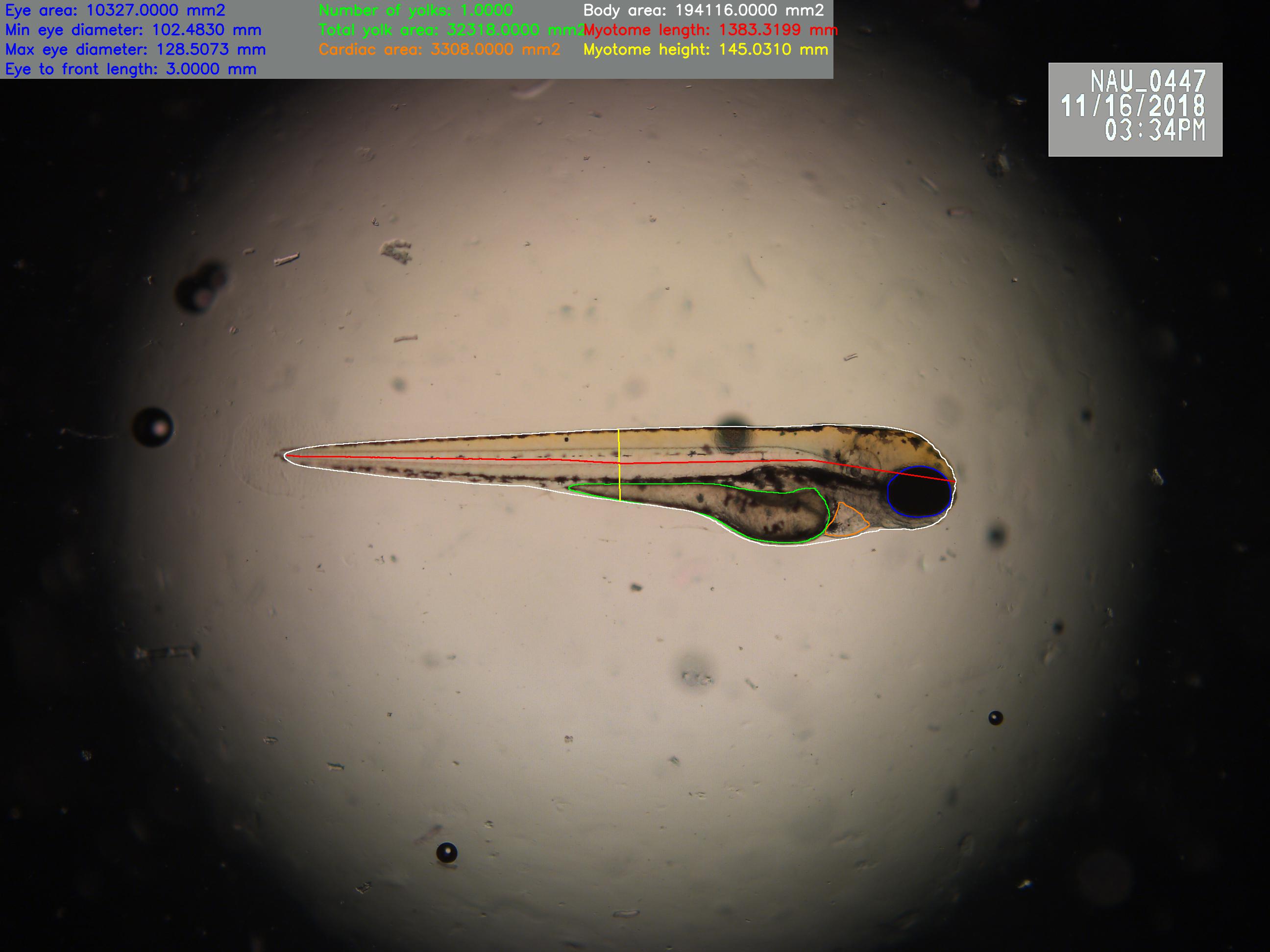


Figure 10 Zebrafish (Danio rerio). Foto: Bjørn Henrik Hansen, SINTEF Ocean


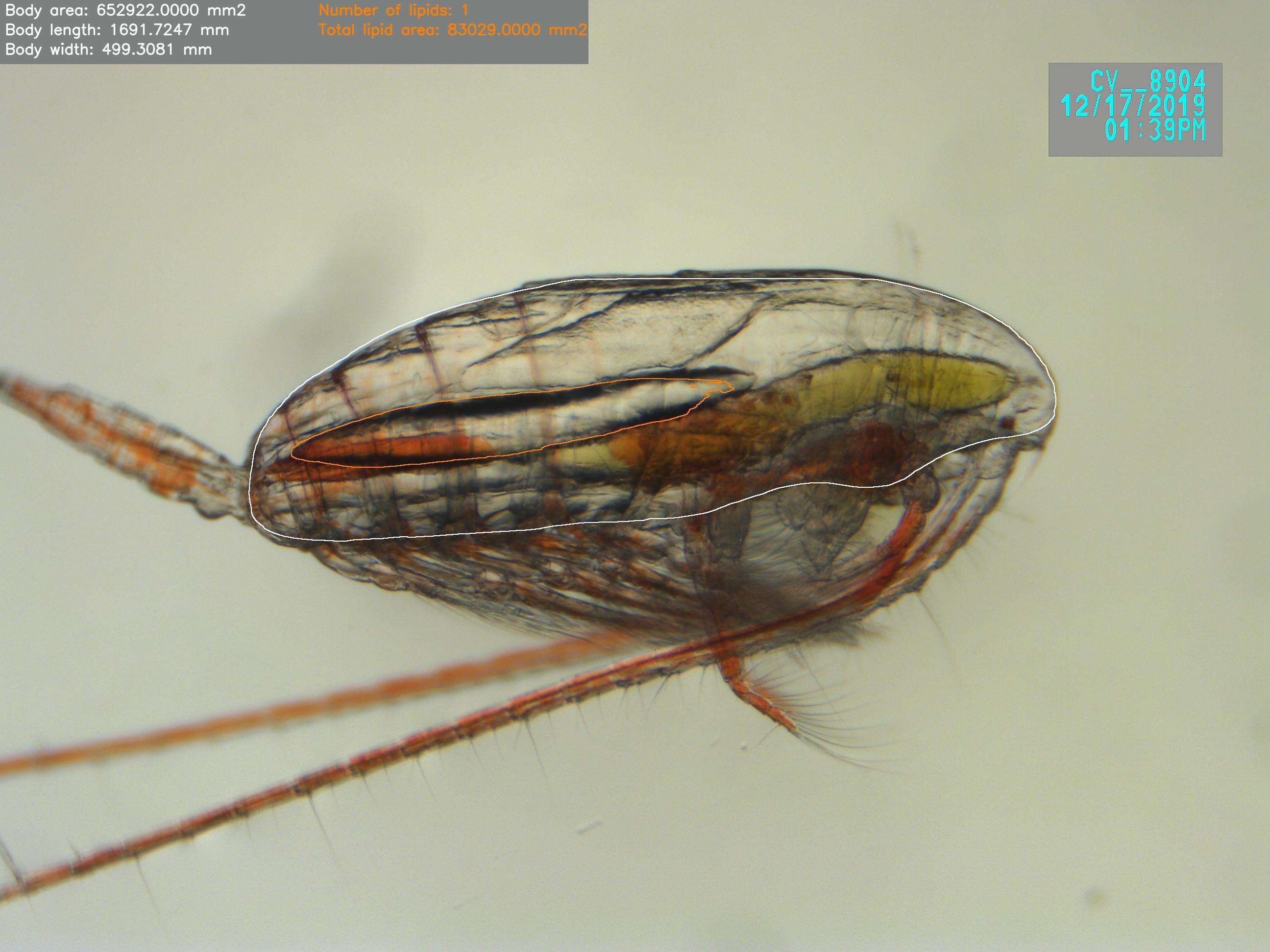


Figure 11 Calanus finmarchicus. Foto: Linn Hjelmeland Svendheim, Nord University
